# Treatment of Children With GH in the United States and Europe: Long-Term Follow-Up From NordiNet^®^ IOS and ANSWER Program

**DOI:** 10.1210/jc.2019-00775

**Published:** 2019-07-15

**Authors:** Lars Sävendahl, Michel Polak, Philippe Backeljauw, Jo Blair, Bradley S Miller, Tilman R Rohrer, Alberto Pietropoli, Vlady Ostrow, Judith Ross

**Affiliations:** 1 Karolinska Institutet, Karolinska University Hospital, Solna, Sweden; 2 Hôpital Necker Enfants Malades, Paris, France; 3 Cincinnati Center for Growth Disorders, Cincinnati Children’s Hospital Medical Center, University of Cincinnati College of Medicine, Cincinnati, Ohio; 4 Alder Hey Children’s NHS Foundation Trust, Liverpool, United Kingdom; 5 University of Minnesota Masonic Children’s Hospital, Minneapolis, Minnesota; 6 University Children’s Hospital, Saarland University Medical Center, Homburg, Germany; 7 Novo Nordisk Health Care AG, Zurich, Switzerland; 8 Novo Nordisk Inc., Plainsboro, New Jersey; 9 Thomas Jefferson University, Philadelphia, Pennsylvania; 10 Nemours/DuPont Hospital for Children, Wilmington, Delaware

## Abstract

**Context:**

Understanding real-world prescribing of GH may help improve treatment of eligible patients.

**Objective:**

Overall: to assess real-world effectiveness and safety of GH (Norditropin). This analysis: to compare clinical characteristics of GH-treated children in the United States and Europe.

**Design:**

The American Norditropin Studies: Web-Enabled Research Program (ANSWER; 2002 to 2016, United States) and the NordiNet International Outcome Study (NordiNet IOS; 2006 to 2016, Europe) were multicenter longitudinal observational cohort studies.

**Setting:**

Data were recorded in 207 (United States) and 469 (Europe) clinics.

**Participants:**

Patients with GH deficiency, Turner syndrome, Noonan syndrome, idiopathic short stature, Prader–Willi syndrome, or born small for gestational age, who commenced GH treatment aged <1 year.

**Intervention:**

GH was prescribed by treating physicians according to local practice.

**Main Outcomes Measures:**

Baseline data and drug doses were recorded. Data on effectiveness and safety were collected.

**Results:**

ANSWER had 19,847 patients in the full analysis set (FAS; patients with birthdate information and one or more GH prescription) and 12,660 in the effectiveness analysis set (EAS; GH-naive patients with valid baseline information). NordiNet IOS had 17,711 (FAS) and 11,967 (EAS). Boys accounted for 69% (ANSWER) and 57% (NordiNet IOS). Treatment start occurred later than optimal to improve growth. The proportion of boys treated was generally larger, children were older at treatment start, and GH doses were higher in the United States vs Europe. No new safety signals of concern were noted.

**Conclusions:**

In most indications, more boys than girls were treated, and treatment started late. Earlier diagnosis of GH-related disorders is needed. The data support a favorable benefit–risk profile of GH therapy in children.

## Teaser

Two large noninterventional studies (ANSWER and NordiNet IOS) examined real-world use of GH across six indications in children. Prescribing differences were found between the United States and Europe.

GH is widely used for the treatment of short children across a number of disorders, primarily with the aim of improving linear growth toward the normal adult range. GH has been approved for the treatment of children with GH deficiency (GHD), born small for gestational age (SGA), or with Turner syndrome (TS), Noonan syndrome (NS), idiopathic short stature (ISS), or chronic renal disease, and for growth failure in Prader–Willi syndrome (PWS).

In addition to evidence from randomized controlled trials of the efficacy and safety of GH, which underpinned the various approvals, several large-scale observational studies of GH treatment (1–6) have provided important supplemental evidence, specifically on GH’s long-term safety. Data from these studies have also made it possible to derive GH response-prediction models (7, 8).

This paper reports results from the NordiNet International Outcome Study (NordiNet IOS; ClinicalTrials.gov no. NCT00960128) and the American Norditropin Studies: Web-Enabled Research Program (ANSWER; NCT01009905), two noninterventional, multicenter studies assessing the effectiveness and safety of real-world treatment with somatropin (Norditropin; Novo Nordisk A/S, Copenhagen, Denmark) in pediatric and adult patients. We report on clinical characteristics of, and GH dosing in, short children with GHD, born SGA, or with TS, NS, ISS, or PWS, followed up for up to 10 years of Norditropin treatment. These are compared between the United States and Europe with the intention that this description of current practices could help to guide future treatment decisions. In particular, sex differences in the frequency of treatment are underlined and discussed.

Effectiveness and safety results for pediatric patients from NordiNet IOS and ANSWER, as well as data on adult patients, will be reported in separate publications. Data from subsets of the patients in the two studies have been published previously (9–15).

## Materials and Methods

The design and methodology of NordiNet IOS and ANSWER have been reported previously (16). Key points are summarized below and in Table 1.

**Table 1.** Design and Key Features of NordiNet IOS and ANSWER

	NordiNet IOS	ANSWER
Study start	1 April 2006	24 June 2002
Study end	31 December 2016	30 September 2016
No. of countries involved (no. of patients per country)	22 [Belgium (11), Czech Republic (1213), Denmark (719), Finland (211), France (3619), Germany (7008), Hungary (86), Ireland (41), Israel (152), Italy (103), Lithuania (103), Luxembourg (2), Montenegro (85), Netherlands (152), Norway (297), Russia (156), Serbia (1299), Slovenia (281), Spain (6), Sweden (941), Switzerland (523), United Kingdom (694)]^*a*^	1 [United States (19,847)]
No. of clinics	469	207
Important protocol amendments	Prior to protocol amendment 3 (30 September 2013), only nonserious ARs and SAEs, regardless of relatedness, MESIs, and pregnancies, were collected. The change to the protocol was due to new pharmacovigilance legislation, which came into effect in July 2012. ARs and SARs considered related to GH, and SAEs considered unrelated to GH, were included in this report	Prior to protocol amendment 2 (9 November 2011), very few events were reported to the study. This is likely to have reflected the way ARs and SARs were described in earlier versions of the protocol and revised text, with amendment 2 more clearly explaining the collection and reporting of safety information. Only events reported after this date were included in this report

Abbreviations: AR, adverse reaction; MESI, medical event of special interest; SAE, serious adverse event; SAR, serious adverse reaction.

^a^ One additional country, Saudi Arabia, participated in the study and registered patients to the International Outcomes database; however, none of those satisfied the inclusion criteria of the study.

NordiNet IOS was a multicenter, longitudinal, observational cohort study based on systematic collection of data using the NordiNet IOS Web-based system. ANSWER was originally a postmarketing registry of adults and pediatric patients in the United States treated with Norditropin, and it was developed into a noninterventional, observational study. The two studies were complementary, with similar aims, using the same electronic platform for data management. Results of the studies are presented side by side, rather than pooled, because the reporting of effectiveness and safety endpoints differed.

In both studies, Norditropin was administered as directed by the treating physician and according to routine practice and local regulations. Approval was obtained from relevant ethics committees, written consent was obtained, and all data were pseudonymized. The studies were conducted in accordance with the Declaration of Helsinki, Guideline for Good Pharmacoepidemiology Practices, and regulatory requirements.

Pediatric patients were eligible for inclusion when they had an appropriate diagnostic condition and when treatment with Norditropin was initiated before 18 years of age. Diagnoses were determined by the participating physicians. For study purposes, patients with more than one diagnosis recorded were classified as follows: patients with a syndrome and coexisting GHD were classified by syndrome; patients recorded as both SGA and GHD (isolated or multiple pituitary hormone deficiency) were classified as GHD.

Baseline data were collected according to routine clinical practice (16). Follow-up time on GH treatment during childhood with known dose (mg/kg/d) was recorded.

In both studies, numerous effectiveness endpoints were assessed (16), with change from baseline in height SD score (SDS) as a key outcome. Data on IGF-I SDS were reported in both studies (NordiNet IOS and ANSWER).

Safety data were based on physicians’ reporting of adverse events (AEs). Adverse reactions (ARs; AEs deemed related to product), classed as serious (SARs) or nonserious (NSARs), were recorded. In NordiNet IOS, serious AEs (SAEs) not related to GH therapy were also recorded throughout the study. (For SAEs in both studies, an SAE was defined as an experience that resulted in any of the following: death, a life-threatening experience, hospitalization or prolongation of existing hospitalization, a persistent or significant disability/incapacity, or a congenital anomaly/birth defect. Important medical events that might not result in death, be life-threatening, or require hospitalization could be considered as SAEs if, based on appropriate medical judgement, they might jeopardize the patient and might require medical or surgical intervention to prevent one of the outcomes listed. The term “life-threatening” in the definition of an SAE referred to an event in which the patient was at risk for death at the time of the event. It did not refer to an event that hypothetically might have caused death if it had been more severe.) In ANSWER, however, it was not mandatory to report SAEs that were not related to GH therapy until 9 November 2011, when the protocol was amended. Fewer than 20 SAEs had been reported prior to this amendment, but subsequently the number increased. Only SAEs reported after that date were included in the analysis. In both studies, a double assessment of causality was used to identify any causal relationship to GH therapy: causality was reported by the treating physician, and all SAEs, including SARs, were also reviewed by the study sponsor. When either the original reporter or the study sponsor considered the relationship as possible or probable, the event was considered as an AR.

### Statistical analysis

In both studies, the effectiveness analysis set (EAS), used to analyze effectiveness outcomes, comprised patients reported as GH-naive at baseline (however, in ANSWER, GH-naive patients could have been treated with GH for up to 6 months before enrollment) and with valid baseline height, age, and dosing information. The full analysis set (FAS), used to evaluate safety outcomes, included all patients with available birthdate information and with at least one Norditropin prescription recorded. Baseline and exposure data were summarized using descriptive statistics based on data available.

## Results

### Patient disposition

**Figure 1.**
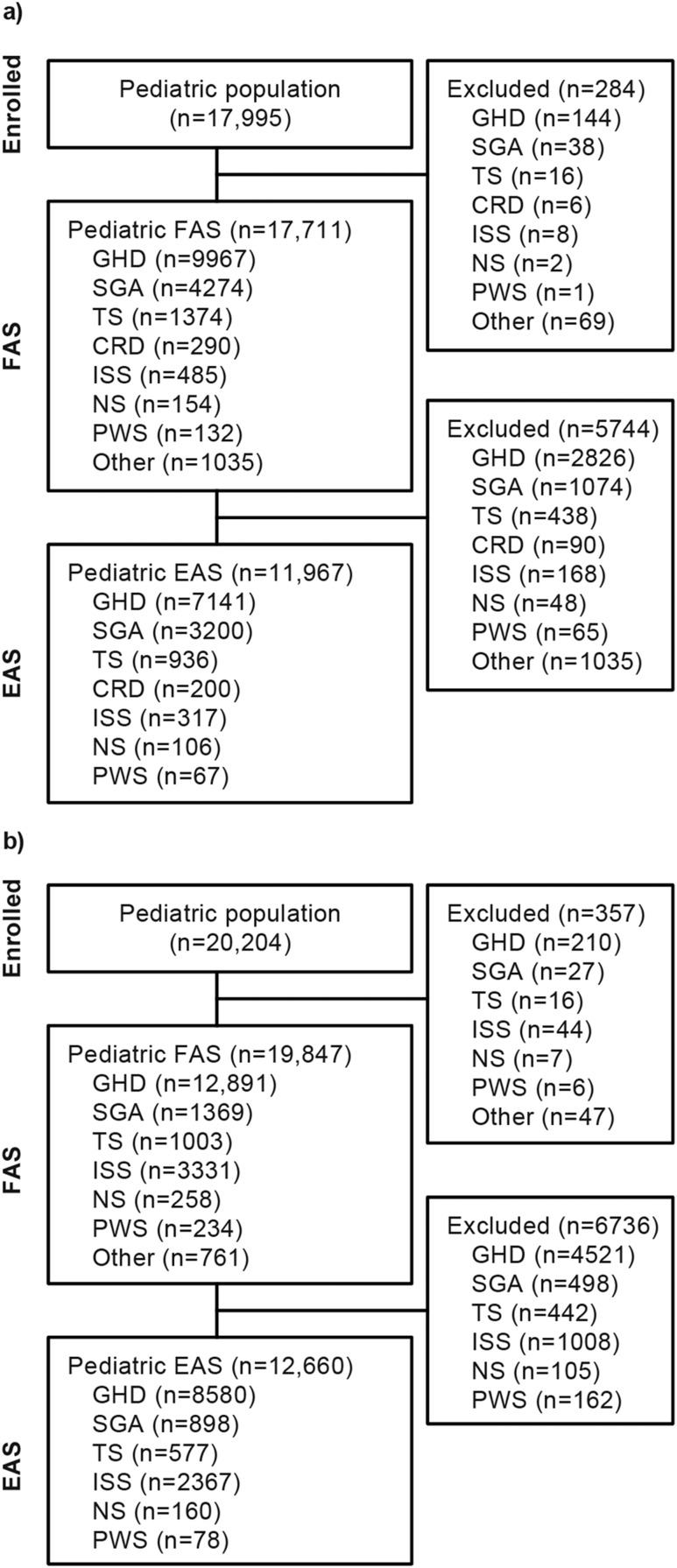
Disposition of patients in (a) NordiNet IOS and (b) ANSWER. CRD, chronic renal disease.

**Figure 2.**
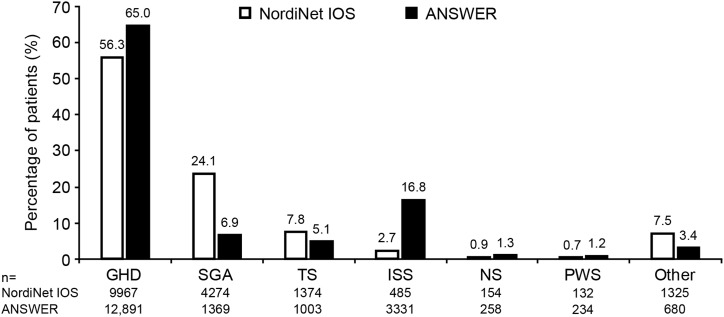
Distribution of diagnostic groups in the two studies (FAS). “Other” indicates other diagnoses and chronic renal disease.

NordiNet IOS enrolled 17,995 pediatric patients, of whom 17,711 were included in the FAS and 11,967 in the EAS (Fig. 1a). The 11,967 in the EAS included 200 patients with chronic renal disease; these patients are not discussed further in this report. ANSWER enrolled 20,204 pediatric patients, of whom 19,847 were included in the FAS and 12,660 in the EAS (Fig. 1b). The most common indications in the FAS in both studies were GHD, ISS, SGA, and TS, with SGA more common in NordiNet IOS and ISS more common in ANSWER (Fig. 2). Enrollment by country is shown in Table 1.

### Patient characteristics at baseline

**Figure 3.**
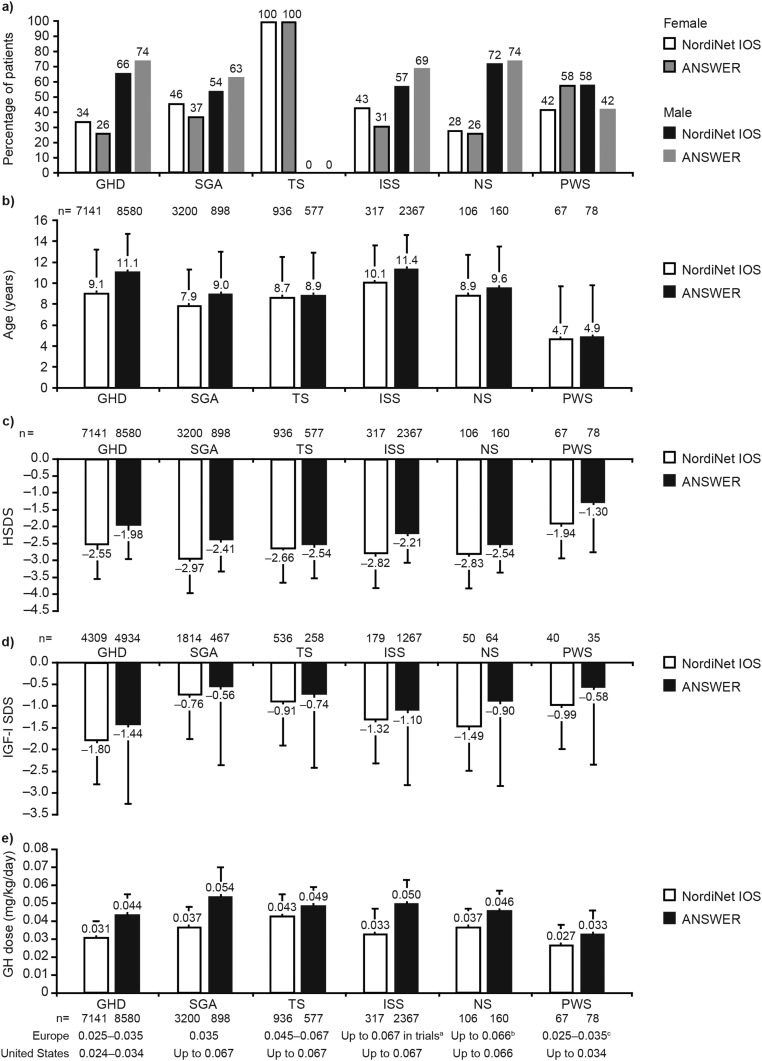
(a) Sex, (b) age at treatment start, (c) height SDS (HSDS), (d) IGF-I SDS, and (e) dose of GH at treatment start by diagnostic group in NordiNet IOS and ANSWER. All data are for patients included in the EAS; data are mean (SD). Approved doses (mg/kg/d) are shown below each diagnostic group. ^a^ISS is not an approved indication in Europe. ^b^Approved in Switzerland and Japan. ^c^Approved in Switzerland.

In both studies, more than half of the patients were boys, with a higher overall proportion of boys than girls in ANSWER (69%) than in NordiNet IOS (57%). Across GHD, SGA, and ISS, proportionally more boys were enrolled in ANSWER than in NordiNet IOS. In both studies, >2.5-fold as many boys as girls with NS were enrolled. For PWS, there were proportionally more girls than boys in ANSWER, but the reverse was true in NordiNet IOS [Table 2 (17); Fig. 3a].

**Table 2.** Baseline Characteristics and GH Exposure of Enrolled Patients in NordiNet IOS and ANSWER

EAS (FAS)	NordiNet IOS											
	GHD		SGA		TS		ISS		NS		PWS	
	7141 (9967)		3200 (4274)		936 (1374)		317 (485)		106 (154)		67 (132)	
	n	Mean (SD)	n	Mean (SD)	n	Mean (SD)	n	Mean (SD)	n	Mean (SD)	n	Mean (SD)
Baseline characteristics												
Female/male, n (%)	7141	2460 (34%)/ 4681 (66%)	3200	1463 (46%)/ 1737 (54%)	936	936 (100%)/0	317	136 (43%)/ 181 (57%)	106	30 (28%)/76 (72%)	67	28 (42%)/39 (58%)
Age at baseline, y	7141	9.12 (4.12)	3200	7.92 (3.37)	936	8.72 (3.75)	317	10.06 (3.54)	106	8.86 (3.80)	67	4.67 (5.00)
Prepubertal, %	6415	84%	2990	88%	868	90%	237	79%	95	84%	58	95%
												
Bone age/chronological age	3453	0.79 (0.22)	1741	0.79 (0.19)	507	0.87 (0.16)	127	0.84 (0.16)	43	0.80 (0.14)	16	0.80 (0.31)
Baseline HSDS^*a*^	7141	−2.55 (1.10)	3200	−2.97 (0.91)	936	−2.66 (0.93)	317	−2.82 (0.99)	106	−2.83 (1.13)	67	−1.94 (1.48)
Target HSDS (national standard)^*a*^	6533	−0.65 (0.98)	2954	−0.94 (0.95)	856	−0.29 (0.95)	281	−0.87 (0.98)	92	−0.67 (0.91)	42	0.32 (0.84)
Baseline IGF-I SDS (Brabant)^*b*^	4309	−1.80 (1.53)	1814	−0.76 (1.45)	536	−0.91 (1.44)	179	−1.32 (1.63)	50	−1.49 (1.15)	40	−0.99 (1.70)
Baseline GH peak, ng/mL	4045	4.72 (4.79)	719	14.49 (12.76)	113	9.64 (10.78)	84	12.90 (9.89)	32	8.39 (4.53)	13	4.41 (5.67)
GH exposure and treatment follow-up												
GH dose at baseline, mg/kg/d	7141	0.031 (0.009)	3200	0.037 (0.011)	936	0.043 (0.012)	317	0.033 (0.014)	106	0.037 (0.010)	67	0.027 (0.011)
GH dose during treatment, mg/kg/d [mean (SD)]	7141	0.032 (0.008)	3200	0.038 (0.009)	936	0.044 (0.009)	317	0.038 (0.014)	106	0.040 (0.009)	67	0.026 (0.008)
GH dose during treatment, mg/kg/d [median (P10; P90)]	7141	0.032 (0.024; 0.041)	3200	0.035 (0.029; 0.049)	936	0.044 (0.033; 0.053)	317	0.034 (0.025; 0.056)	106	0.039 (0.031; 0.052)	67	0.027 (0.015; 0.035)
Treatment follow-up, y	7141	3.80 (2.87)	3200	3.64 (2.79)	936	4.32 (2.81)	317	3.33 (2.40)	106	3.40 (2.93)	67	4.02 (3.46)
Treatment follow-up, y [median (P10; P90)]	7141	3.30 (0.48;7.74)	3200	3.21 (0.00; 7.66)	936	3.89 (0.87; 8.47)	317	2.95 (0.71; 6.70)	106	2.75 (0.28; 6.77)	67	3.06 (0.65; 9.38)
PYE (FAS)^*c*^	n	PYE	n	PYE	n	PYE	n	PYE	n	PYE	n	PYE
	9967	39,967	4274	16,213	1374	6045	485	1737	154	597	132	662

EAS (FAS)	ANSWER											
	GHD		SGA		TS		ISS		NS		PWS	
	8580 (12,891)		898 (1369)		577 (1003)		2367 (3331)		160 (258)		78 (234)	
	n	Mean (SD)	n	Mean (SD)	n	Mean (SD)	n	Mean (SD)	n	Mean (SD)	n	Mean (SD)
Baseline characteristics												
Female/male, n (%)	8580	2195 (26%)/ 6385 (74%)	898	334 (37%)/ 564 (63%)	577	577 (100%)/0	2367	735 (31%)/ 1632 (69%)	160	41 (26%)/119 (74%)	78	45 (58%)/33 (42%)
Age at baseline, y	8580	11.09 (3.61)	898	9.00 (3.98)	577	8.92 (4.03)	2367	11.38 (3.17)	160	9.61 (3.93)	78	4.91 (4.88)
Prepubertal, %	7141	69%	794	80%	551	89%	2008	65%	132	80%	68	88%
Bone age/ chronological age	4004	0.89 (0.15)	476	0.87 (0.16)	245	0.90 (0.24)	1298	0.90 (0.135)	72	0.86 (0.24)	15	1.04 (0.17)
Baseline HSDS (CDC)^*d*^	8580	−1.98 (0.98)	898	−2.41 (0.92)	577	−2.54 (0.99)	2367	−2.21 (0.86)	160	−2.54 (0.82)	78	−1.30 (1.46)
Target HSDS (CDC)^*d*^	7440	−0.42 (0.99)	795	−0.72 (0.96)	490	0.03 (1.03)	2118	−0.69 (0.92)	138	−0.36 (0.94)	65	−0.07 (1.01)
Baseline IGF-I SDS (Brabant)^*b*^	4934	−1.44 (1.81)	467	−0.56 (1.80)	258	−0.74 (1.68)	1267	−1.10 (1.72)	64	−0.90 (1.94)	35	−0.58 (1.77)
Baseline GH peak, ng/mL	3077	6.39 (4.71)	NR	NR	NR	NR	NR	NR	NR	NR	NR	NR
GH exposure and treatment follow-up												
GH dose at baseline, mg/kg/d	8580	0.044 (0.011)	898	0.054 (0.016)	577	0.049 (0.010)	2367	0.050 (0.013)	160	0.046 (0.011)	78	0.033 (0.013)
GH dose during treatment, mg/kg/d [mean (SD)]	8580	0.046 (0.012)	898	0.054 (0.015)	577	0.049 (0.010)	2367	0.051 (0.013)	160	0.048 (0.011)	78	0.031 (0.012)
GH dose during treatment, mg/kg/d [median (P10; P90)]	8580	0.044 (0.034; 0.061)	898	0.053 (0.038; 0.071)	577	0.049 (0.038; 0.059)	2367	0.049 (0.039; 0.068)	160	0.046 (0.035; 0.064)	78	0.031 (0.018; 0.047)
Treatment follow-up, y	8580	2.20 (15.46)	898	2.24 (2.01)	577	3.05 (2.36)	2367	2.06 (6.02)	160	2.6 (2.0)	78	3.33 (3.12)
Treatment follow-up, y [median (P10; P90)]	8580	2.06 (0.28; 5.30)	898	1.62 (0.27; 5.06)	577	2.52 (0.40; 6.47)	2367	1.68 (0.20; 4.93)	160	2.23 (0.49; 5.94)	78	2.51 (0.26; 8.00)
PYE (FAS)^*c*^	n	PYE	n	PYE	n	PYE	n	PYE	n	PYE	n	PYE
	12,891	33,253	1369	3403	1003	3157	3331	7667	258	707	234	847

EAS pertains except where FAS is also shown. Values are mean (SD) unless otherwise stated.

Abbreviations: HSDS, height SDS; NR, not reported; P10/P90, 10th/90th percentile; PYE, patient-years of exposure.

^a^ For NordiNet IOS, HSDS and target HSDS were calculated using age- and sex-specific national references.

^b^ IGF-I SDS was calculated using the Brabant *et al*. (17) 2003 age- and sex-specific reference (Nichols advantage).

^c^ Total PYE were 70,436 (NordiNet IOS) and 52,070 (ANSWER).

^d^ For ANSWER, HSDS and target HSDS were calculated using the U.S. Centers for Disease Control and Prevention growth tables available at www.cdc.gov/growthcharts.

Sex distribution was examined by age (≥3 vs <3 years) and by GH peak at baseline (<7 or ≥7 ng/mL) for patients with GHD to determine whether it was influenced by these parameters. In both studies, the proportion of male patients was greater in older compared with younger children (Table 3), but there was no difference in sex distribution by GH peak (Table 3).

**Table 3.** Sex Distribution by Age and GH Peak at Baseline for Patients With GHD in NordiNet IOS and ANSWER (EAS)

	Age at Baseline		
	<3 y	≥3 y	Total
NordiNet IOS			
Female, n (% within group)	227 (40.8)	2233 (33.9)	2460 (34.4)
Male, n (% within group)	329 (59.1)	4352 (66.0)	4681 (65.5)
Total, n (% of all patients)	556 (7.8)	6585 (92.2)	7141 (100.0)
ANSWER			
Female, n (% within group)	126 (37.2)	2069 (25.1)	2195 (25.5)
Male, n (% within group)	212 (62.7)	6173 (74.8)	6385 (74.4)
Total, n (% of all patients)	338 (3.9)	8242 (96.1)	8580 (100.0)

	GH Peak at Baseline			
	<7 ng/mL	≥7 ng/mL	Missing	Total
NordiNet IOS				
Female, n (% within group)	1093 (32.7)	238 (33.8)	1129 (36.4)	2460 (34.4)
Male, n (% within group)	2249 (67.2)	465 (66.1)	1967 (63.5)	4681 (65.5)
Total, n (% of all patients)	3342 (46.8)	703 (9.8)	3096 (43.4)	7141 (100.0)
ANSWER				
Female, n (% within group)	457 (24.9)	296 (23.7)	1442 (26.2)	2195 (25.5)
Male, n (% within group)	1373 (75.0)	951 (76.2)	4061 (73.7)	6385 (74.4)
Total, n (% of all patients)	1830 (21.3)	1247 (14.5)	5503 (64.1)	8580 (100.0)

Values are for EAS.

Information on pubertal status was available for >82% of patients in all indications in both studies, with one exception (ISS in NordiNet IOS –75%) (numbers are shown in Table 2). In both studies, most patients for whom information was available were prepubertal (generally >80% in both studies, and >65% for GHD and ISS in ANSWER) (Table 2). For all indications, treatment started at an older age in ANSWER compared with NordiNet IOS (Table 2; Fig. 3b). Excluding PWS, mean age at treatment start ranged from 8 to 10 years in NordiNet IOS and 9 to 11 years in ANSWER. In both studies, patients with ISS were the oldest and patients with PWS were youngest when compared with other indications, followed by SGA in NordiNet IOS and TS in ANSWER.

Across indications, children were shorter at treatment start in NordiNet IOS than in ANSWER. In NordiNet IOS, patients born SGA were the shortest at baseline (height SDS almost −3) (Table 2; Fig. 3c). In ANSWER, patients with TS or NS were the shortest. With the exception of PWS, where treatment is based on body composition rather than height, patients with GHD were the tallest of those starting GH treatment in both studies. In ANSWER, the mean height SDS for patients with GHD was only just within the cut-off for short stature (height SDS less than −2).

Mean IGF-I SDS was <0 at baseline for all indications in both studies, and lower in NordiNet IOS than in ANSWER (Fig. 3d).

### Exposure to GH and duration of treatment follow-up

For all indications, doses of GH at treatment start were higher in the United States than in Europe (Table 2; Fig. 3e). In both studies, mean starting doses at baseline were well within the approved doses for approved indications [Table 4 (18, 19)].

**Table 4.** Approved Doses of Norditropin (mg/kg/d)

	Europe	United States
GHD	0.025–0.035 mg/kg/d or 0.7–1.0 mg/m^2^ BSA	0.024–0.034 mg/kg/d
SGA	0.035 mg/kg/d or 1.0 mg/m^2^ BSA	Up to 0.067 mg/kg/d
TS	0.045–0.067 mg/kg/d or 1.3–2.0 mg/m^2^ BSA	Up to 0.067 mg/kg/d
ISS	Not approved^*a*^	Up to 0.067 mg/kg/d
NS	Up to 0.066 mg/kg/d (Switzerland and Japan)	Up to 0.066 mg/kg/d
PWS	0.025–0.035 mg/d (Switzerland)	0.034 mg/kg/d
CRD	0.050 mg/kg/d or 1.4 mg/m^2^ BSA	Not approved

See (18, 19) for information on Norditropin doses.

Abbreviations: BSA, body surface area; CRD, chronic renal disease.

^a^ 0.033–0.067 mg/kg/d has been used in trials.

For most indications, mean overall dose during follow-up was similar to mean dose at baseline; however, mean dose increased slightly during follow-up for patients with NS and ISS in NordiNet IOS (Table 2).

In all indications, duration of treatment follow-up was longer in NordiNet IOS than in ANSWER (Table 2), reflecting the fact that many patients in ANSWER were enrolled near the end of the study. Patients in ANSWER may also have been switched to other GH brands due to reimbursement issues.

### Concomitant medications

Information on concomitant medications did not have to be reported and was available for only 39.1% (7757/19,847) of patients in the FAS in ANSWER and 10.6% (1869/17,711) of patients in the FAS in NordiNet IOS. Among these patients, treatments for hypothyroidism (mainly levothyroxine) were commonly prescribed. The proportions of patients prescribed treatments for hypothyroidism, as a percentage of all patients in the FAS, were as follows in ANSWER: GHD 14.5%, SGA 6.5%, TS 18.9%, ISS 6.9%, NS 6.6%, and PWS 20.1%. In NordiNet IOS, proportions were as follows: GHD 7.7%, SGA 1.9%, TS 7.9%, ISS 3.5%, NS 2.6%, and PWS 1.5%.

In the United States, methylphenidate and dexmethylphenidate were prescribed for attention deficit hyperactivity disorder (ADHD) as follows: for patients with GHD 9.1%, SGA 6.7%, TS 6.9%, ISS 8.3%, NS 13.2%, and PWS 4.3%. These two drugs were prescribed to 8.6% of patients in the whole cohort (all indications). No prescriptions for methylphenidate were recorded in Europe, but other ADHD drugs may have been prescribed.

### Safety endpoints

Patient-years of exposure (PYE), based on the FAS, are shown in Table 2. Total PYE were 70,436 (NordiNet IOS) and 51,070 (ANSWER).

Detailed safety results for pediatric patients from NordiNet IOS and ANSWER will be reported in a separate publication, but are summarized here. The most frequent events across indications were those commonly observed in children treated with GH and included headache, injection-site reactions, edema, and arthralgia. For each indication, the safety profile was consistent with data in the approved labeling for Norditropin; no new safety signals of concern were observed.

In NordiNet IOS, 421 ARs were reported in 334 pediatric patients: 288 NSARs in 249 patients and 133 SARs in 90 patients. There were also 352 SAEs reported in 224 patients. There were 11 deaths among pediatric patients; causality was considered “unlikely” by both the reporter and the study sponsor in all but two cases (Table 5). For two patients, both with multiple comorbidities, the reporter considered a causal link with GH was “possible” whereas the study sponsor considered a causal link unlikely; narratives are provided in Table 6.

**Table 5.** Overview of Fatal Cases by Indication in NordiNet IOS and ANSWER

Indication	Age Group at Onset (y)^*a*^	Sex	Duration of GH Treatment (y)	Event Onset (Years After Start of GH Treatment)^*b*^	Event Reported as Fatal	Causality^*c*^
NordiNet IOS						
GHD	Adolescent	M	Unknown	3.02	Cardiac failure	Unlikely
GHD	Infant	F	Unknown	0.87	Cardiac failure	Unlikely
GHD	Child	F	0.9	0.89	Acute myeloid leukemia	Unlikely
GHD	Adolescent	M	7.9	8.9	Metastases to meninges	Reporter: possible; Novo Nordisk: unlikely
SGA	Child	F	7.0	7.00	Cardiac failure	Unlikely
CRD	Adult	M	Unknown	8.79	Cerebral hemorrhage; subdural hematoma; brain edema; cerebral venous thrombosis; transverse sinus thrombosis; depressed level of consciousness	Unlikely
CRD	Child	M	Unknown	2.28	Pulmonary edema	Unlikely
PWS	Adult	M	0.7	0.70	Sudden unexpected death	Unlikely
Other diagnoses	Child	F	Unknown	1.91	Sepsis; bone marrow depression bleeding (hemorrhage); bone marrow depression bleeding (bone marrow failure)	Unlikely
Other diagnoses	Child	M	0.6	0.53	Medulloblastoma recurrent	Unlikely
Other diagnoses	Adolescent	M	0.3, then discontinued for 0.8, then treated for 0.8	0.25	Primitive neuroectodermal tumor; condition aggravated	Unlikely
ANSWER						
GHD	Infant	F	Unknown	0.5	Respiratory distress	Unlikely
GHD	Adolescent	M	5.8	5.8	Cause unknown	Unlikely
GHD	Child	F	1.7 (approx.)	1.7 (approx..)	Child abuse	Unlikely
PWS	Child	F	2.0	2.0	Cardiac arrest	Unlikely

Abbreviation: CRD, chronic renal disease.

^a^ Defined as: infant, aged <2 y; child, aged 2–12 y; adolescent, aged 13–19 y; adult, aged ≥ 20 y.

^b^ Time is shown to the onset of the event. Deaths occurred on the same date or later.

^c^ Causality is shown for reporter and Novo Nordisk. Causality was the same for both unless listed in the table.

**Table 6.** Narratives for Two Fatal Cases in NordiNet IOS

Event	Narrative
Event reported as fatal: metastases to meninges	An adolescent male in the GHD group, treated with GH for 8 y. Medical history included: suprasellar cyst, hydrocephalus, short stature, congenital neurocutaneous melanosis, and known hydrocephalus. He became increasingly tired, shaky, and complained about abdominal pain; GH treatment was withdrawn. One year after the last GH injection, the patient was diagnosed with leptomeningeal melanocytosis and neurologic symptoms due to leptomeningeal tumor. The patient died 9.6 mo later.
Events reported as fatal: brain edema, cerebral hemorrhage, cerebral venous thrombosis, depressed level of consciousness, subdural hematoma, and transverse sinus thrombosis	An adult male, treated from childhood with GH due to childhood-onset GHD and CRD for an unknown length of time. Medical history included: panhypopituitarism due to empty sella syndrome, GHD, perinatal asphyxia, CRD with peritoneal dialysis, and epilepsy. He was admitted to the emergency department with reduced consciousness, with subsequent collapse and shock requiring endotracheal intubation, and died 2 wk later.

For both cases, causality was considered as “possible” by the reporter and “unlikely” by Novo Nordisk.

Abbreviation: CRD, chronic renal disease.

In ANSWER, 396 ARs overall were reported in 284 pediatric patients: 328 NSARs in 240 patients and 68 SARs in 49 patients. Among 81 patients, 128 SAEs were reported after 9 November 2011. Death was reported as an SAE in four patients; causality was judged to be “unlikely” in all cases (Table 5).

### IGF-I SDS during follow-up

In both studies, for most indications, mean IGF-I SDS increased to >0 by year 1 (EAS and FAS). For all indications, mean values remained >0 at each year of follow-up in NordiNet IOS, and at most years in ANSWER.

## Discussion

These long-term data from NordiNet IOS and ANSWER, based on 70,436 and 51,070 PYE, respectively, provide information on characteristics of patients prescribed Norditropin across six different indications. In the United States compared with Europe, the proportion of boys was larger in all indications except PWS, patients were generally older and taller at treatment start, and doses of GH were higher at baseline and throughout follow-up.

Our data confirm previous reports that more males than females are treated with GH in GHD, ISS, and SGA (1–4). The question of whether biology or sociocultural differences underlie the sex imbalance has been discussed extensively, notably recently by Ranke *et al.* (4). Our data suggest that the tendency to refer more boys than girls is more common after the age of 3 years. No impact of baseline GH peak (<7 or ≥7 ng/mL) on referral patterns by sex was observed, suggesting that boys were not more or less GH deficient at referral than girls. Limitations of these data are that only a small proportion of our cohort was aged <3 years, and that stimulation test results were available for only 57% of GHD patients in NordiNet IOS and 36% in ANSWER.

In all indications except PWS, the sex difference was more highly skewed toward males in the United States compared with Europe, suggesting that at least some of the sex imbalance in the United States arises from factors other than biology. Similar United States–European differences for GHD and SGA were reported in recent analyses from the Kabi International Growth Study (KIGS) (4) and from GeNeSiS (2).

Across all indications, patients were generally older at treatment start in ANSWER than in NordiNet IOS, in line with findings from KIGS (4) for GHD, ISS, and SGA. In contrast, Pfäffle *et al.* (2) reported that age at treatment start was similar between the United States and Germany, but higher across indications in France. Overall, data from these different analyses suggest that, in general, mean age at treatment start was higher than desired. Several studies, including previous subanalyses of ANSWER and NordiNet IOS (12, 14, 20, 21), have reported that starting treatment earlier rather than later is associated with better growth response.

National surveillance systems, algorithm-based data mining of electronic medical records, and provision of tools for parents to monitor their children’s growth could help to detect growth failure earlier. For example, earlier diagnosis and referrals to specialist care increased in Finland after an automated growth-monitoring strategy was integrated into the primary care electronic health record system (22). Educational programs should specifically target the need for physicians and families to be aware of short stature or decreased growth in girls.

For all indications, children were taller at treatment start in ANSWER compared with NordiNet IOS, possibly because, in ANSWER, children could already have had up to 6 months of GH treatment before enrollment, or because children were generally older at treatment start compared with NordiNet IOS. Furthermore, childhood GHD is a multifaceted condition that may require treatment even in the absence of short stature (23).

A limitation of all international observational studies is that local differences in the diagnosis of conditions and access to treatment could confound the results. Marked United States–European differences were observed in the sizes of the study populations in some indications. The smaller SGA population and larger ISS population in the United States compared with Europe could result from the later approval of Norditropin in the United States for SGA (2008) vs Europe (2004). Alternatively, children in Europe may have been classified as SGA rather than ISS to qualify for treatment, as Norditropin is not licensed for ISS in Europe. However, as concerns regarding safety of GH in short children born SGA (24) have been raised by some but not by others, it is important to classify SGA correctly, as was done by Sävendahl *et al.* (15), and to analyze safety outcomes carefully (25).

In all indications, and as expected due to differences in prescribing practice, GH doses at baseline and during treatment were higher in the United States compared with Europe. Mean GH starting doses were expected to fall within the approved range, and this was the case for all indications except for GHD in the United States. Low mean starting doses for PWS reflect the young ages of the children, the approved dose in the United States, and the fact that, as PWS is not an approved indication in many countries in Europe, it may have been dosed in accordance with GHD dosing.

In Europe, doses were noticeably below approved doses for TS and NS. The low dose for NS may reflect possible prescription by clinicians at GHD-approved doses, as within Europe NS is only approved in Switzerland, which is not under European Medicines Agency jurisdiction. Previous reports from NordiNet IOS have noted that GH dosing varied considerably across indications and countries, with patients with TS frequently receiving doses below the approved level (10, 13).

It is reassuring that no new safety signals of concern were noted in any of the indications. Data from a number of large-scale studies of GH treatment, covering all indications reported in the individual studies, were recently reviewed (26). The authors concluded that the data provide reassurance on the long-term safety of GH treatment, but that patients with preexisting risk factors for diabetes and malignancy should be treated with caution and monitored regularly during follow-up.

The limited data available on concomitant medications showed that stimulant medications were prescribed in the United States at a frequency (8.6%) similar to that in children without growth disorders (reported as 5.1% based on the 2016 National Survey of Children’s Health) (27). A subanalysis of ANSWER data reported that the linear growth response to GH treatment was not modified by ADHD stimulant medication (28); however, in a subanalysis of KIGS data, Miller *et al.* concluded that the ADHD phenotype, alone or in conjunction with stimulant therapy, may impair the short-term growth response to GH treatment (29).

As mentioned above, NordiNet IOS and ANSWER are subject to the limitations of large, multicenter, observational studies. During the period covered (2002 to 2016), there were changes in diagnostic practices and definitions of eligibility for GH treatment, in particular with respect to GHD diagnosis, and changes in approvals for indications and recommended doses. Changes in prescribing practices due to financial constraints or external influences are likely to have occurred and to have differed by country. Comorbidities, concomitant medications, and AEs may have been underreported due to protocol amendments or reported differently according to local practice. Indeed, possible underreporting of AEs has been acknowledged as a limitation in similar registries such as GeNeSIS (6) and KIGS (30).

However, the two similar studies in different geographic regions provide a wealth of real-world data, enabling comparison of treatment practices between the United States and Europe in NS and PWS, in addition to indications previously compared in other studies (2, 4, 5). The data describe the characteristics of patients that clinicians could expect to meet in everyday clinical practice, and they highlight areas where improvements could be considered, such as earlier identification of patients and, in some cases, adjustments to GH dosing. The results also provide reassurance regarding the safety of GH, especially because no increased safety risks were evident in data from the United States, where consistently higher doses of GH were used. The populations are more heterogeneous than those from single-region studies, and the larger numbers available will facilitate analyses of the safety of GH therapy in indications such as NS, where reports to date have been limited to small numbers.

In conclusion, results from ANSWER and NordiNet IOS show that, at treatment start, across all indications, mean age was higher and children were taller, and mean doses of GH at baseline and during treatment were higher, in the United States than in Europe. In both regions, diagnoses, and therefore treatment start, occurred later than optimal to improve growth. In most indications, more boys were treated than girls, with the sex difference greater in the United States than in Europe for GHD, ISS, and SGA. Overall, these data accrued from 37,558 pediatric patients and 121,506 PYE support the favorable benefit–risk profile of treatment with Norditropin across the indications analyzed.

## Abbreviations:

ADHD: attention deficit hyperactivity disorder
AE: adverse event
ANSWER: American Norditropin Studies: Web-Enabled Research Program
AR: adverse reaction
EAS: effectiveness analysis set
FAS: full analysis set
GHD: GH deficiency
ISS: idiopathic short stature
KIGS: Kabi International Growth Study
NordiNet IOS: NordiNet International Outcome Study
NS: Noonan syndrome
NSAR: nonserious AR
PYE: patient-years of exposure
PWS: Prader–Willi syndrome
SAE: serious AE
SAR: serious AR
SDS: SD score
SGA: small for gestational age
TS: Turner syndrome

## References

[1] Kaplowitz PB, Shulman DI, Frane JW, Jacobs J, Lippe B Characteristics of children with the best and poorest first- and second-year growth during rhGH therapy: data from 25 years of the Genentech national cooperative growth study (NCGS). Int J Pediatr Endocrinol. 2013;2013(1):9. 23631505 10.1186/1687-9856-2013-9 PMC3660178

[2] Pfäffle R, Land C, Schönau E, Holterhus PM, Ross JL, Piras de Oliveira C, Child CJ, Benabbad I, Jia N, Jung H, Blum WF Growth hormone treatment for short stature in the USA, Germany and France: 15 years of surveillance in the Genetics and Neuroendocrinology of Short-Stature International Study (GeNeSIS). Horm Res Paediatr. 2018;90(3):169–180. 30199857 10.1159/000492397

[3] Plotnick L, Rapaport R, Desrosiers P, Fuqua JS Update from the GHMonitorSM observational registry in children treated with recombinant human growth hormone (Saizen). Pediatr Endocrinol Rev. 2009;6(Suppl 2):278–282. 19337182

[4] Ranke MB, Lindberg A, Tanaka T, Camacho-Hübner C, Dunger DB, Geffner ME Baseline characteristics and gender differences in prepubertal children treated with growth hormone in Europe, USA, and Japan: 25 Years’ KIGS^®^ experience (1987–2012) and review. Horm Res Paediatr. 2017;87(1):30–41. 27915352 10.1159/000452887

[5] Wyatt D Lessons from the national cooperative growth study. Eur J Endocrinol. 2004;151(Suppl 1):S55–S59. 15339245 10.1530/eje.0.151s055

[6] Child CJ, Zimmermann AG, Chrousos GP, Cummings E, Deal CL, Hasegawa T, Jia N, Lawrence S, Linglart A, Loche S, Maghnie M, Pérez Sánchez J, Polak M, Predieri B, Richter-Unruh A, Rosenfeld RG, Yeste D, Yorifuji T, Blum WF Safety outcomes during pediatric GH therapy: final results from the prospective GeNeSIS observational program. J Clin Endocrinol Metab. 2019;104(2):379–389. 30219920 10.1210/jc.2018-01189 PMC6300411

[7] Ranke MB, Lindberg A; KIGS International Board. Prediction models for short children born small for gestational age (SGA) covering the total growth phase. Analyses based on data from KIGS (Pfizer International Growth Database). BMC Med Inform Decis Mak. 2011;11(1):38. 21627853 10.1186/1472-6947-11-38 PMC3125313

[8] Ranke MB, Lindberg A, Chatelain P, Wilton P, Cutfield W, Albertsson-Wikland K, Price DA Derivation and validation of a mathematical model for predicting the response to exogenous recombinant human growth hormone (GH) in prepubertal children with idiopathic GH deficiency. KIGS International Board. Kabi Pharmacia International Growth Study. J Clin Endocrinol Metab. 1999;84(4):1174–1183. 10199749 10.1210/jcem.84.4.5634

[9] Blankenstein O, Pedersen BT, Schlumpf M, Andreasen AH, Júlíusson PB Management and interpretation of heterogeneous observational data: using insulin-like growth factor-I data from the NordiNet^®^ International Outcome Study. Growth Horm IGF Res. 2015;25(1):41–46. 25542446 10.1016/j.ghir.2014.12.001

[10] Blankenstein O, Snajderova M, Blair J, Pournara E, Pedersen BT, Petit IO Real-life GH dosing patterns in children with GHD, TS or born SGA: a report from the NordiNet^®^ International Outcome Study. Eur J Endocrinol. 2017;177(2):145–155. 28522645 10.1530/EJE-16-1055 PMC5488395

[11] Lee PA, Ross JL, Pedersen BT, Kotnik P, Germak JA, Christesen HT Noonan syndrome and Turner syndrome patients respond similarly to 4 years’ growth-hormone therapy: longitudinal analysis of growth-hormone-naïve patients enrolled in the NordiNet^®^ International Outcome Study and the ANSWER Program. Int J Pediatr Endocrinol. 2015;2015(1):17. 26351466 10.1186/s13633-015-0015-1 PMC4562101

[12] Polak M, Blair J, Kotnik P, Pournara E, Pedersen BT, Rohrer TR Early growth hormone treatment start in childhood growth hormone deficiency improves near adult height: analysis from NordiNet^®^ International Outcome Study. Eur J Endocrinol. 2017;177(5):421–429. 28780521 10.1530/EJE-16-1024 PMC5633042

[13] Polak M, Konrad D, Tønnes Pedersen B, Puras G, Šnajderová M Still too little, too late? Ten years of growth hormone therapy baseline data from the NordiNet^®^ International Outcome Study. J Pediatr Endocrinol Metab. 2018;31(5):521–532. 29652668 10.1515/jpem-2017-0489

[14] Ross J, Lee PA, Gut R, Germak J Factors influencing the one- and two-year growth response in children treated with growth hormone: analysis from an observational study. Int J Pediatr Endocrinol. 2010;2010(1):494656. 20981140 10.1155/2010/494656 PMC2957130

[15] Sävendahl L, Pournara E, Pedersen BT, Blankenstein O Is safety of childhood growth hormone therapy related to dose? Data from a large observational study. Eur J Endocrinol. 2016;174(5):681–691. 26903552 10.1530/EJE-15-1017

[16] Höybye C, Sävendahl L, Christesen HT, Lee P, Pedersen BT, Schlumpf M, Germak J, Ross J The NordiNet^®^ International Outcome Study and NovoNet^®^ ANSWER Program^®^: rationale, design, and methodology of two international pharmacoepidemiological registry-based studies monitoring long-term clinical and safety outcomes of growth hormone therapy (Norditropin^®^). Clin Epidemiol. 2013;5:119–127. 23658497 10.2147/CLEP.S42602 PMC3641810

[17] Brabant G, von zur Mühlen A, Wüster C, Ranke MB, Kratzsch J, Kiess W, Ketelslegers JM, Wilhelmsen L, Hulthén L, Saller B, Mattsson A, Wilde J, Schemer R, Kann P; German KIMS Board. Serum insulin-like growth factor I reference values for an automated chemiluminescence immunoassay system: results from a multicenter study. Horm Res. 2003;60(2):53–60. 12876414 10.1159/000071871

[18] Novo Nordisk. Norditropin prescribing information. Available at: www.novo-pi.com/norditropin.pdf. Accessed 15 November 2018.

[19] Novo Nordisk. Norditropin summary of product characteristics. Available at: www.medicines.org.uk/emc/medicine/2760. Accessed 15 November 2018.

[20] Ross J, Lee PA, Gut R, Germak J Impact of age and duration of growth hormone therapy in children with Turner syndrome. Horm Res Paediatr. 2011;76(6):392–399. 22156541 10.1159/000333073

[21] Ross JL, Lee PA, Gut R, Germak J Increased height standard deviation scores in response to growth hormone therapy to near-adult height in older children with delayed skeletal maturation: results from the ANSWER Program. Int J Pediatr Endocrinol. 2015;2015(1):1. 25904938 10.1186/1687-9856-2015-1 PMC4405836

[22] Sankilampi U, Saari A, Laine T, Miettinen PJ, Dunkel L Use of electronic health records for automated screening of growth disorders in primary care. JAMA. 2013;310(10):1071–1072. 24026604 10.1001/jama.2013.218793

[23] Growth Hormone Research Society. Consensus guidelines for the diagnosis and treatment of growth hormone (GH) deficiency in childhood and adolescence: summary statement of the GH Research Society. J Clin Endocrinol Metab. 2000;85(11):3990–3993. 11095419 10.1210/jcem.85.11.6984

[24] Carel JC, Ecosse E, Landier F, Meguellati-Hakkas D, Kaguelidou F, Rey G, Coste J Long-term mortality after recombinant growth hormone treatment for isolated growth hormone deficiency or childhood short stature: preliminary report of the French SAGhE study. J Clin Endocrinol Metab. 2012;97(2):416–425. 22238382 10.1210/jc.2011-1995

[25] Albertsson-Wikland K, Mårtensson A, Sävendahl L, Niklasson A, Bang P, Dahlgren J, Gustafsson J, Kriström B, Norgren S, Pehrsson NG, Odén A Mortality is not increased in recombinant human growth hormone-treated patients when adjusting for birth characteristics. J Clin Endocrinol Metab. 2016;101(5):2149–2159. 26918292 10.1210/jc.2015-3951

[26] Stochholm K, Kiess W Long-term safety of growth hormone-A combined registry analysis. Clin Endocrinol (Oxf). 2018;88(4):515–528. 29055168 10.1111/cen.13502

[27] Danielson ML, Bitsko RH, Ghandour RM, Holbrook JR, Kogan MD, Blumberg SJ Prevalence of parent-reported ADHD diagnosis and associated treatment among U.S. children and adolescents, 2016. J Clin Child Adolesc Psychol. 2018;47(2):199–212. 29363986 10.1080/15374416.2017.1417860 PMC5834391

[28] Rose SR, Reeves G, Gut R, Germak J Attention-deficit/hyperactivity disorder medication treatment impact on response to growth hormone therapy: results from the ANSWER program, a non-interventional study. J Pediatr. 2015;167(6):1389–1396. 26394822 10.1016/j.jpeds.2015.08.036

[29] Miller BS, Aydin F, Lundgren F, Lindberg A, Geffner ME Stimulant use and its impact on growth in children receiving growth hormone therapy: an analysis of the KIGS International Growth Database. Horm Res Paediatr. 2014;82(1):31–37. 24924157 10.1159/000360005

[30] Darendeliler F, Karagiannis G, Wilton P Headache, idiopathic intracranial hypertension and slipped capital femoral epiphysis during growth hormone treatment: a safety update from the KIGS database. Horm Res. 2007;68(Suppl 5):41–47. 18174706 10.1159/000110474

