# CORRIGENDUM for “Treatment of Children With GH in the United States and Europe: Long-Term Follow-Up From NordiNet® IOS and ANSWER Program”

**DOI:** 10.1210/clinem/dgaa139

**Published:** 2020-04-23

In the above-named article by Sävendahl L, Polak M, Backeljauw P, Blair J, Miller B, Rohrer T, Pietropoli A, Ostrow V, and Ross J (*J Clin Endocrinol Metab.* 2019;104(10):4730–4742; doi: 10.1210/jc.2019-00775) the “Participants” paragraph of the abstract should read:

**“Participants:** Patients with GH deficiency, Turner syndrome, Noonan syndrome, idiopathic short stature, Prader-Willi syndrome, or born small for gestational age, who commenced GH treatment aged <18 years.”

